# Do Domestic Dogs (*Canis lupus familiaris*) Perceive Numerosity Illusions?

**DOI:** 10.3390/ani10122304

**Published:** 2020-12-04

**Authors:** Miina Lõoke, Lieta Marinelli, Carla Jade Eatherington, Christian Agrillo, Paolo Mongillo

**Affiliations:** 1 Laboratory of Applied Ethology, Department of Comparative Biomedicine and Food Science, University of Padua, Piazzetta del Donatore, 4, 35020 Legnaro, Italy; miina.looke@studenti.unipd.it (M.L.); carlaeatherington12@gmail.com (C.J.E.); paolo.mongillo@unipd.it (P.M.); 2 Department of General Psychology, University of Padua, 35131 Padua, Italy; christian.agrillo@unipd.it; 3 Padua Neuroscience Center, University of Padua, 35131 Padua, Italy

**Keywords:** quantity, dog, numerical cognition, gestalt, illusion, vision, solitaire

## Abstract

**Simple Summary:**

Studying visual illusions in animals allows researchers to reveal similarities and differences between how human and non-human species perceive the world around them. Recently, investigations into dogs have found evidence for the differential perception of visual illusions, when compared with human observers. Here, we extended this line of investigation by testing dogs’ susceptibility to numerosity illusions. This type of illusion occurs when an individual under- or overestimates the number of objects presented in a visual scene owing to the spatial arrangement of the objects. In the current study, we observed the spontaneous likelihood for dogs to approach a larger quantity of food items. In Experiment 1, we first established whether dogs would try to maximize their food intake within the experimental context. Following this, Experiments 2 and 3 presented food items arranged so as to generate a well-known numerosity illusion—the Solitaire illusion. Overall, dogs were able to select the larger quantity of food (Experiment 1), but did not exhibit any evidence of a numerosity misperception in Experiments 2 and 3. Our results reinforce the idea that dogs’ representation of the world differs significantly from ours.

**Abstract:**

Recent studies have showed that domestic dogs are only scantly susceptible to visual illusions, suggesting that the perceptual mechanisms might be different in humans and dogs. However, to date, none of these studies have utilized illusions that are linked to quantity discrimination. In the current study, we tested whether dogs are susceptible to a linear version of the Solitaire illusion, a robust numerosity illusion experienced by most humans. In the first experiment, we tested dogs’ ability to discriminate items in a 0.67 and 0.75 numerical ratio. The results showed that dogs’ quantity discrimination abilities fall in between these two ratios. In Experiment 2, we presented the dogs with the Solitaire illusion pattern using a spontaneous procedure. No evidence supporting any numerosity misperception was found. This conclusion was replicated in Experiment 3, where we manipulated dogs’ initial experience with the stimuli and their contrast with the background. The lack of dogs’ susceptibility to the Solitaire illusion suggests that numerical estimation of dogs is not influenced by the spatial arrangement of the items to be enumerated. In view of the existing evidence, the effect may be extended to dogs’ quantitative abilities at large.

## 1. Introduction

Understanding the subjective world of animals has interested philosophers and scientists for a long time. Neurobiological investigation of the retina and of the neural circuits supporting vision has advanced our knowledge regarding the type of information captured by the eye and how this information is processed in the brain. However, neurobiological investigation is almost blind with respect to the overall perception of an individual. For instance, in the presence of ambiguous patterns, like the ‘Arcimboldo faces’, establishing whether an animal perceives a face or a mix of fruits and vegetables by the mere analysis of neural responses to these stimuli is not possible. To address this issue, behavioural studies using visual illusions are largely adopted in the field of cognitive ethology [1,2,3]. The investigation of visual illusions allows an understanding of the perceptual mechanisms underlying the perception of colour, shapes, orientation, and motion of objects [4,5]. Illusions also represent a useful tool to compare the perception among vertebrates, a fact that helps researchers to assess how similar or dissimilar the subjective worlds of different species are. These types of studies showed that chimpanzees are susceptible to the Delboeuf illusion [6]; proved rhesus monkeys to perceive the Zöllner illusion [7] as well as the rotating snake illusion [8]; while capuchin monkeys perceive the Müller–Lyer illusion [9] and the Delboeuf illusion [10]. Taken together, the investigation of visual illusions in apes and monkeys has led researchers to hypothesize that human and non-human primates share at least partially similar perceptual systems.

**Figure 1:**
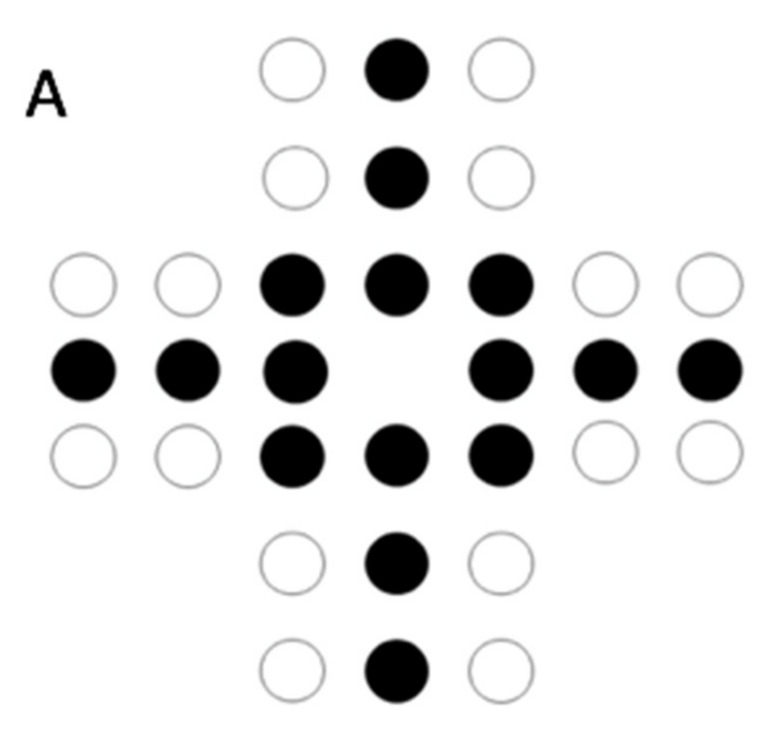

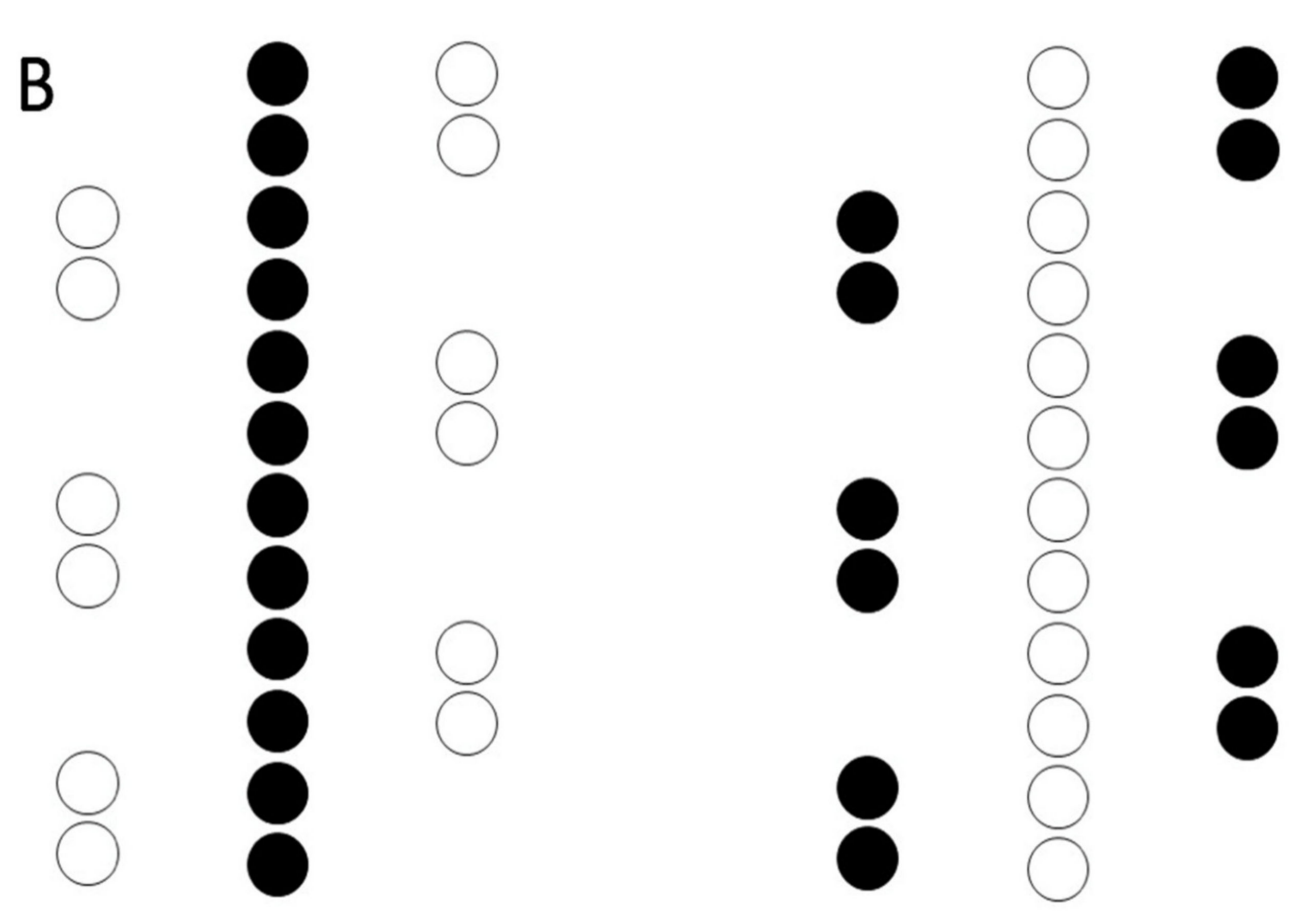
The original Solitaire illusion array (**A**) and the linear version of the Solitaire illusion used in Experiments 2 and 3. Black dots represent inedible items and white dots represent food items (**B**).

Numerosity illusions refer to a class of visual illusions where we tend to misperceive the number of objects included in the visual scene because their spatial arrangement. For instance, in the Solitaire illusion, the number of centrally-located items is typically overestimated compared with items located in the perimeter in separate, smaller clusters (Figure 1A) [11]. The illusion appears to be based on the Gestalt principles of proximity and good continuation, whereby we tend to perceive items that are respectively close to one another, and in a straight line, as part of the same object [12], with perceptual features that are different from the mere sum of its constituent parts. Unlike many other illusory phenomena, this illusion has never been convincingly found in non-human primates. Our closest relatives, chimpanzees, do not show any evidence of susceptibility to this numerosity illusion, even if tested with two different experimental paradigms [13]. Rhesus monkeys do not perceive the illusion either [13], while data from capuchin monkeys are mixed, with two studies showing a slight illusory effect only at the population level [13,14]. However, larger inter-individual variability has been observed in the studies of capuchin monkeys compared with the human literature [15,16], suggesting a weaker perception of this illusion. A recent study using alternative (linear) versions of the Solitaire illusion (Figure 1B) led to the same conclusions in both rhesus and capuchin monkeys [17]. This robust body of evidence led researchers to advance the hypothesis that human-unique physical experiences of the world cause us to have difficulties when forming accurate estimations of numerosity [14]. Extending the investigation of the Solitaire illusions in other species would be fundamental to addressing this issue.

In the last decade, there has been an increasing number of studies on visual illusions in non-primate species; for recent reviews, see Feng et al. [1] and Agrillo et al. [2]. Dogs, in particular, have been attracting the attention of researchers given the differential perception of many illusory phenomena compared with human observers. Dogs, for instance, are not susceptible to the Delboeuf illusion [18,19] or the Müller–Lyer illusion [20]. Weak evidence in favour of a human-like perception was provided by two studies using the Ponzo illusion [21,22], as well as in a recent study using the Ehrenstein illusory contour illusion [23]. In the case of Ebbinghaus illusion [19], dogs even exhibited a reversed illusion, perceiving as larger the object typically seen as smaller by human observers. It is worth noting that all these illusory patterns are related to size misperception or illusory contours. To date, although there are multiple demonstrations of quantitative abilities in dogs [24,25,26,27,28,29,30,31,32], no study has investigated their susceptibility to numerosity illusions—without which we cannot understand to what extent the subjective worlds of dogs and humans are different.

In the current study, we investigated whether dogs are susceptible to the Solitaire illusion. As a recent study showed that items located on the periphery are perceived as 76% more numerous compared with centrally located items by humans [15], in the first experiment, we assessed dogs’ quantitative skills in the ratios of 0.67 (8 vs. 12 food pieces) and 0.75 (9 vs. 12). Although previous studies revealed that the quantity discrimination abilities of untrained dogs fall in a lower range, such experiments were performed with smaller numerosities than the ones typically used in the Solitaire illusion. However, quantity discrimination thresholds are known to be affected by set size [33]. Thus, it seemed necessary to assess dogs’ quantitative abilities with a set size comparable to the one later used to assess their susceptibility to the Solitaire illusion. Indeed, in Experiment 2, we tested dogs’ susceptibility to the Solitaire illusion using food items arranged in two arrays, reproducing a linear version (Figure 1B) of the illusion made by Frith and Frith [11]. In one array, food items (*n* = 12) were centrally located, while plastic stickers (*n* = 12) were located in the perimeter. In the other array, the same number of food items were located in the perimeter and were split in two clusters by centrally located plastic stickers. If dogs were susceptible to the Solitaire illusion, they were expected to select the array in which the food items were centrally located. Lastly, to ensure that the results of Experiment 2 were not significantly affected by the experimental procedure adopted, we replicated it by manipulating dogs’ initial experience of the stimuli and their contrast with the background (Experiment 3).

## 2. Experiment 1

The first experiment aimed to establish whether dogs display sufficient quantitative discrimination abilities to possibly perceive the illusory ratio generated in humans by the Solitaire pattern. Dogs were presented with two numerical ratios: 0.67 (8 vs. 12) and 0.75 (9 vs. 12). These ratios are close to the threshold of numerical acuity of many vertebrate species [34,35,36,37,38]. Unlike the previous literature on dogs’ spontaneous quantity discrimination abilities [25,26,28], we used larger numerosities, similar to the ones used in the illusionary pattern.

### 2.1. Methods

#### 2.1.1. Subjects

The sample consisted of forty pet dogs (26 females and 14 males, mean age ± SD = 5.2 ± 3.6 years) recruited through the University of Padua’s Laboratory of Applied Ethology database of volunteers. The criteria for recruitment were that dogs were motivated by food and in good health. Eleven dogs were mixed breeds and the remainder were purebred of various dog breeds.

Dogs were randomly divided into two equally sized groups (*n* = 20), each of which was presented with a different discrimination ratio, 0.67 and 0.75. There was no significant age difference between the two groups (*t*-test; t = 1.396; *p* = 0.171). One group consisted of 8 males and 12 females and the other of 6 males and 14 females.

#### 2.1.2. Stimuli

The stimuli consisted of two plates, where different numbers of sausage slices were placed. Plates were white, round ceramic plates, with a diameter of 18.5 cm. Each sausage slice had a diameter of 16 mm and a thickness of 3 mm. During the procedure, two plates were simultaneously presented to the dog, one of which had 12 pieces of sausage on it, while the other had either 8 or 9 pieces. Therefore, two different pairs of stimuli could be presented, one with a ratio of 0.67 (8 and 12 pieces), and one with a ratio of 0.75 (9 and 12 pieces). There is evidence that, in the presence of two groups of food items, dogs spontaneously reached the larger group in an attempt to maximize their food intake [25,26,27]. Therefore, we expected that, if dogs were able to discriminate between 8 (or 9) and 12 pieces of food, they would have selected the larger group. To reduce the possibility that the spatial arrangement influenced dogs’ choices, the sausage slices were pseudo-randomly arranged on the plate with two constraints: (1) the pieces could not be closer to each other than 15 mm, and (2) the slices were spread across the plate in a way that they covered the whole area of the plate, without overt biases towards the centre or the edges of the plate. The side where the larger amount was presented was randomised and counterbalanced across all dogs within each group.

#### 2.1.3. Experimental Setting and Procedure

**Figure 2:**
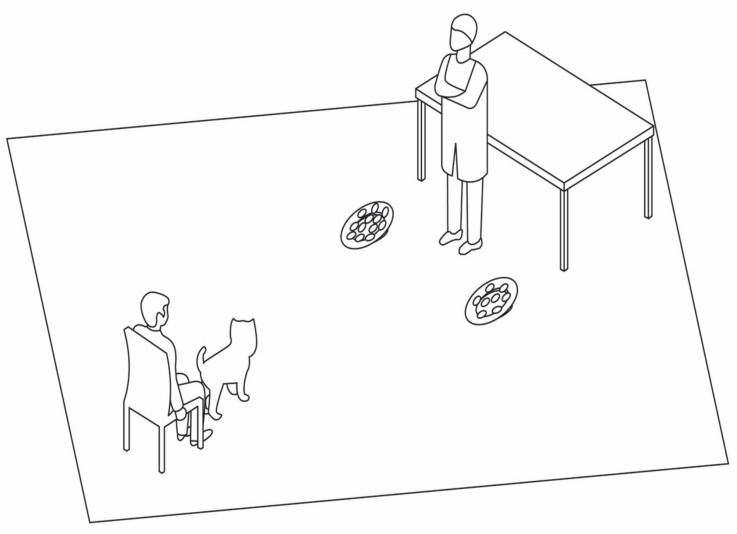
The experimental setting in Experiment 1.

The experiment was conducted in a quiet room (Figure 2) measuring 4.7 × 5.8 m. The room was equipped with a table where the plates were placed before the presentation; two wooden stands in front of the table to keep the plates at a 10° angle towards the dog during the presentation; a chair for the owner facing the stands from a distance of 1.5 m; and two cameras (Xacti VPC-WH1, Sanyo, Moriguchi, Japan) to record the experiment, one facing the dog and the other facing the experimenter.

The dog was brought into the testing room by the owner and let off leash for 5 min to become acquainted with the area, while the experimenter gave instructions to the owner. Following this, the experimenter presented the dog with a single plate with three sausage slices on it. The rationale for this preliminary presentation was to allow dogs to experience that they could eat food from the plates, as well as to ascertain that dogs were food-motivated and comfortable to get close enough to the experimenter to retrieve food. The plate was presented centrally in between the two stands to avoid creating any bias towards one side. Following this presentation, the owner was asked to leave the room together with the dog, while the experimenter prepared stimuli for the actual test and placed the plates on the table.

When re-entering the room, the owner was instructed to sit and keep the dog in between their legs in a marked location, with their body facing the experimenter; other than this, owners were instructed not to interfere with the dogs’ orientation at any time. The experimenter stood facing the dog at a distance of 1.5 m, wearing sunglasses to minimise any cues inadvertently given to the dog. The experimenter picked up both plates from the table simultaneously, while maintaining her frontal orientation towards the dog, and called the dog’s name in order to attract their attention. Then, she placed the plates on the stands, 1 m apart from each other. Immediately after placing the plates, she brought her hands together at her chest to avoid giving any cues to the dog. While looking at the ground, she mentally counted to 5 s, then said “OK!”. Upon hearing this signal, the owner was instructed to release the dog. The dogs were allowed to eat the food off the plate they first approached, while the other plate was quickly removed by the experimenter. The approached plate was considered to be the dog’s choice.

#### 2.1.4. Data Collection and Analyses

The data regarding dogs’ choices were collected during the test as a binary variable, as a choice of the larger or smaller quantity. A two-tailed binomial test was then run separately for the 0.67 and the 0.75 ratios (*n* = 20 for each of the two conditions), in order to test the null hypothesis (H_0_) that dogs’ choices were not different from chance level.

Behavioural data were collected and extracted from videos using the Observer XT software (version 12.5, Noldus, Groeningen, The Netherlands). A continuous sampling method was used to collect data about dogs’ head orientation (right plate, left plate, elsewhere) from the moment the experimenter picked the plates up from the table, and thus the plates became visible to the dog, to the moment in which the dog started moving. From the data collected, two variables were obtained, (a) the total time spent looking at either plate and (b) the percentage of such time spent looking at the plate with the larger food amount.

In order to test if the data collected were reliable, inter-observer reliability was assessed on data collected by a second, independent observer. The latter collected data about the dogs’ choices (from the videos taken during testing) from 38 subjects (videos from two dogs were missing because of technical problems). Inter-observer reliability for dog’s head orientation was assessed on a randomly selected subset of videos (25% of the total number of videos).

If the binomial test detected a group-level significant choice for either the 0.67 or the 0.75 condition, a binary logistic regression model was run to test whether dogs looking behaviour explained their choices with that specific ratio. Specifically, we tested if the dog’s choice depended on how much dogs looked at either plate before choosing, or on how such attention was allocated between the two plates. In the model, the dependent variable was the dog’s choice and the independent variables were the total time spent looking at either plate and the percentage of time spent looking at the plate holding the larger food amount.

All statistical analyses were conducted using R [39], with the statistical significance level set at 0.05.

### 2.2. Results

All 40 dogs approached one of the two plates immediately after being released during the test. Overall, out of the 40 dogs, 23 dogs chose the larger quantity. Out of the 20 dogs that were presented with the 0.67 ratio, 17 (85%) chose the plate with the larger quantity and 3 chose the plate with the smaller quantity (two-tailed binominal test; *p* = 0.0026). Of the 20 dogs that were presented with the 0.75 ratio, 6 (30%) chose the plate with the larger quantity and 14 dogs (70%) chose the plate with the smaller quantity (two-tailed binomial test; *p* = 0.1153).

The interobserver reliability for dogs’ choices resulted in 100% agreement in between the two observers. Data collected by the two observers regarding the dogs’ head orientation were highly correlated (Pearson’s correlation; looking at the right plate: r = 0.92, *p* < 0.001, looking at the left plate: r = 0.87, *p* < 0.001, looking elsewhere: r = 0.81, *p* = 0.004).

During the stimuli presentation of the 0.67 ratio, the dogs spent on average 3.2 ± 1.1 s looking at either plate. Out of this time, the dogs spent on average 54.3 ± 22.8% looking at the plate with the larger quantity. The logistic regression model for the 0.67 ratio revealed that neither total looking time (Z = 0.63, *p* = 0.53) nor percentage of looking at the larger amount (Z = 0.72, *p* = 0.47) had a significant effect in explaining dogs’ choices. During the presentation of the 0.75 ratio, the dogs spent on average 3.8 ± 1.4 s looking at either plate and, out of this time, the dogs spent on average 50.4 ± 14.6% looking at the plate with the larger quantity.

### 2.3. Discussion

We observed the spontaneous ability of dogs to select the larger group of food items. At the group level, dogs proved able to spontaneously discriminate between two food quantities with a ratio of 0.67 (8 vs. 12 food pieces), but not between quantities with a ratio of 0.75 (9 vs. 12). In humans, the Solitaire illusion creates a perceived quantitative difference close to the ratio of 0.76 [15]. In view of this, at least some dogs might display sufficient quantitative abilities to be tested in the presence of the Solitaire illusion, which we assessed in Experiments 2 and 3.

That said, the results of Experiment 1 are interesting per se, as they provide new insights into dogs’ quantitative skills. As far as we are aware, this is the first evidence of spontaneous quantitative judgments of dogs in the presence of a relatively large numbers of items. Previous studies have been limited to a smaller number of items (e.g., 2 vs. 3 or 3 vs. 4) [25,26,27,28]. However, a recent study by Rivas-Blanco and colleagues [32] explored dogs’ numerical abilities with larger numerosities and found that the abilities of trained dogs reach up to a ratio of 0.83. Furthermore, the same study highlighted that, when given the possibility, dogs rely on continuous quantities, rather than numerosity. Because, in the current study, we did not control for the continuous quantities, it is possible that the dogs relied on those perceptual cues rather than numerosity. As expected, the discriminative ability showed ratio dependency, as a higher accuracy was observed with a 0.67 ratio than with a 0.75 ratio. This aligns with a large number of studies showing that the precision of quantitative judgements among vertebrates increases when the ratio between the smaller and the larger quantity decreases, in agreement with Weber’s law [37,40,41]. In both conditions, dogs approached the plates immediately when being released. They used much of the time before being released looking at the plates and divided their attention equally between the two stimuli. Along with the lack of effects of attention on dogs’ choices, these suggest that the task conditions were sufficient for the dogs to collect information about the plates and that the procedure is well suited for assessing dogs’ spontaneous choice behaviour.

One may argue that dogs solved the task using olfactory information. However, dogs moved immediately after being released, and went straight to the chosen plate, without expressing any overt olfactory search behaviour. Moreover, Miletto Petrazzini and Wynne [26], using a similar free choice task, showed that dogs presented with an easier ratio (i.e., 0.33) are unable to locate the larger amount of food solely using olfactory cues. Furthermore, previous evidence shows that dogs are unable to spontaneously discriminate food quantities based on olfactory cues, even when presented with quantities with larger differences than those used in the present study, and in spite of being allowed to closely inspect both stimuli before making a choice [42]. It is thus unlikely that dogs based their choice on olfactory cues.

## 3. Experiment 2

In this experiment, we used a procedure similar to that employed in Experiment 1 in order to assess if dogs are susceptible to the Solitaire illusion.

### 3.1. Methods

#### 3.1.1. Subjects

The sample consisted of twenty naïve pet dogs (13 females and 7 males, mean age ± SD = 5.2 ± 3.6 years). Six dogs were mixed breeds and the remainder belonged to various breeds. All dogs were recruited through the University of Padua’s Laboratory of Applied Ethology database of volunteers and the criteria for recruitment were that dogs were motivated by food and in general good health.

#### 3.1.2. Stimuli

The stimuli represented a linear version of the Solitaire illusion [11,17] and consisted of two plates, both with 12 slices of sausage on them (Figure 1B). On one plate, the sausage slices were arranged in a straight, vertical line in the middle of the plate, with a distance of 2 mm between each slice. Besides sausage slices, the stimuli also contained black stickers as non-edible elements, which were chosen in order to make them clearly identifiable as non-food. The twelve inedible black stickers were arranged in groups of two at the sides of this middle line. The distance between the middle line of sausage pieces and the lateral lines of stickers was 4 cm. The other plate had the opposite arrangement, i.e., the middle line consisted of 12 black stickers, while 12 sausage slices were arranged at both sides of the middle line in groups of two.

Plates were light grey rectangular (20 × 30 cm) ceramic plates for human use. As in the previous experiment, sausage slices had a diameter of 16 mm and thickness of 3 mm.

#### 3.1.3. Experimental Setting and Procedure

The experimental setting and the procedure for the presentation of the stimuli were identical to those of the first experiment. However, in the preliminary presentation, the presented plate featured three sausage slices and three stickers, which allowed dogs to experience that the stickers were inedible.

#### 3.1.4. Data Collection and Analyses

The data regarding dogs’ choices were collected during the test as a binary variable. A two-tailed binomial test was then run to test the null hypothesis (H_0_) that dogs’ choices were not different from a chance level. A second observer collected the data about the dogs’ choices from all the videos to perform the reliability analyses. Similar to Experiment 1, no effect of dogs’ looking behaviour on dogs’ accuracy was found, and no data about orientation were collected.

### 3.2. Results

All 20 dogs readily made a choice when presented with the plates. Seven dogs (35%) out of 20 chose the plate with centrally located food items. A two-tailed binominal test revealed that dogs’ choices were not different from chance level (*p* = 0.26). The interobserver reliability resulted in an agreement of 100% between the two observers.

### 3.3. Discussion

In this experiment, we explored whether dogs are susceptible to a linear version of the Solitaire illusion. We accordingly arranged items in a Solitaire illusion pattern that, in humans, is known to generate a solid overestimation of quantity [11]. Dogs chose at a level not significantly different from chance, which suggests that they are not susceptible to the illusion. However, some other explanations must be considered.

One possible reason for dogs choosing at chance level in this experiment was a lack of sufficient knowledge about the different elements of the stimuli (i.e., sausage slices and stickers). As the experiment relied on the dogs’ motivation to reach the largest amount of food, it was crucial they understood that the black stickers were inedible. To this aim, dogs had been given the possibility to explore a plate that contained both stickers and sausages, which we assumed would give them enough information about the stimuli to discriminate them in the actual test. It is possible, however, that such experience was insufficient for them to learn that black stickers were inedible.

Moreover, the colour of the stickers was chosen to make them clearly identifiable; however, this also made them slightly more salient than the sausage slices. It is possible that dogs’ choices were biased owing to the contrast difference between the food and black stickers over the plate background, which made the latter more salient than the food.

To ensure that dogs’ limited experience and the stimuli’s salience were not affecting the results, a third experiment was conducted.

## 4. Experiment 3

This experiment assessed dogs’ susceptibility to the same linear arrangement of the Solitaire illusion assessed in Experiment 2 with a similar spontaneous choice test. However, Experiment 3 started with a training phase aiming to teach dogs to choose a single edible item over an inedible item based only on the visual information. Only after successfully reaching a learning criterion were dogs presented with the test stimuli, featuring the Solitaire illusion arrangement. In addition, black stickers were substituted with blue dots, and grey plates with black panels, in order to equalise the contrast of both food and inedible elements on the background.

### 4.1. Methods

#### 4.1.1. Subjects

The initial sample consisted of 35 naïve pet dogs (19 females and 16 males, mean age ± SD = 5.6 ± 3.1 years). Eleven dogs were mixed breeds and the remainder were purebred dogs of various breeds. All dogs were recruited through the University of Padua’s Laboratory of Applied Ethology database of volunteers and the criteria for recruitment were that dogs were motivated by food and in good health. Out of the initial 35 dogs, 20 dogs (11 females and 9 males, mean age ± SD = 5.0 ± 2.8 years) reached the test phase.

#### 4.1.2. Stimuli

The stimuli consisted of panels with an arrangement of food items and non-food items on them. Panels were square shaped (30 × 30 cm) and made from black polycarbonate. Sausage slices were used as food items and blue dots made out of ethylene-vinyl acetate foam sheet were used as non-food items. Both elements had a diameter of 16 mm and a thickness of 3 mm.

**Figure 3:**
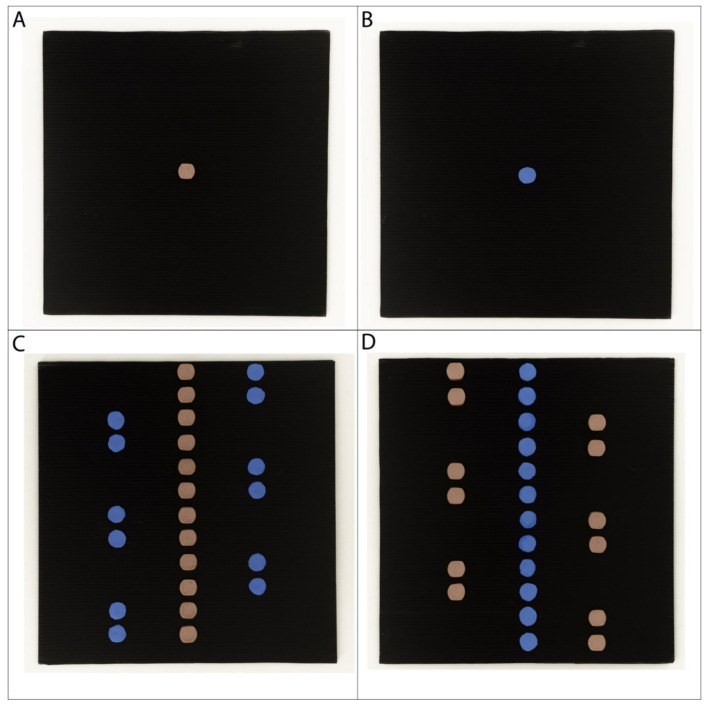
Photographs of the training stimuli (**A**,**B**) and the test stimuli (**C**,**D**) of Experiment 3.

For the training phase, a panel with one food item in the middle served as the positive stimulus (Figure 3A), while a panel with one blue dot served as the negative stimulus (Figure 3B). To control for odour cues, a slice of sausage was placed behind the negative stimulus, out of the dogs’ sight.

The test stimuli represented a linear version of the Solitaire illusion [11,17], with the same arrangement as that used in Experiment 2. One panel had the central line composed of sausage slices (Figure 3C) and the other panel had the central line composed of blue dots (Figure 3D).

#### 4.1.3. Experimental Setting and Procedure

The experimental setting was almost identical to that of Experiments 1 and 2. The only exception was a 1.3 m high barrier in between the table and the stands, in order to prevent the dogs from seeing the panels being prepared during the training phase.

The procedure consisted of a preliminary presentation, a training phase, and a single test trial featuring the illusionary stimuli. The preliminary presentation was identical to that described in Experiments 1 and 2, but the stimulus was a panel with a single sausage slice in the middle; this phase was intended to verify dogs’ motivation to reach the experimenter and eat food out of the panel. The following training phase involved several presentations of the training stimuli, each carried out as described for the single test presentation of Experiments 1 and 2, except the outcome after the dog’s choice: in two initial trials (warmup trials, counterbalanced for stimulus presentation side), if the dog approached the positive stimulus, it was allowed to eat the food, and then encouraged to inspect the panel with the blue dot; if it chose the negative stimulus, it was allowed to inspect it, and then encouraged to explore and eat the food off the positive one. In the following training trials, dogs were allowed to eat the food only if they chose the positive stimulus, after which they were taken back to the starting position; if they chose the negative stimulus, they were immediately taken back for the next trial. A maximum of 15 trials were performed, in each of which the presentation side of the stimulus was counterbalanced according to a pseudorandomised sequence, with the constraint that a stimulus could not be presented on the same side for more than two consecutive trials. If the dog chose the positive stimulus in four consecutive training trials, the phase was considered successfully completed, and the dog was presented with a single test trial. If the criterion was not reached within the 15 trials, the dog underwent no further testing.

The presentation of the test trial was identical to the training trials, but the test stimuli with an illusory configuration, rather than training stimuli, were presented. The presentation side of the stimuli was counterbalanced across all dogs.

#### 4.1.4. Data Collection and Analyses

The number of training trials undertaken by each dog was counted during the experiment. The data regarding dogs’ choices in the test trials were collected during the test as a binary variable. A two-tailed binomial test was then run to test the null hypothesis (H_0_) that dogs’ choices were not different from a chance level.

To assess interobserver reliability, a second independent observer collected data about dogs’ choices in the test trials and the number of training trials undertaken by each dog in the training phase from all the videos.

### 4.2. Results

Twenty out of 35 dogs successfully passed the training phase and were presented with the test stimuli. The average number of training trials ± SD undertaken by those dogs was 8.9 ± 3.2 trials. The minimum number of training trials was 4 and the maximum was 15 trials. The remaining 15 dogs did not choose the panel with food four times in a row within 15 training trials, and thus were not presented with the test stimuli.

In the test trial, 9 (45%) out of 20 dogs chose the stimulus with centrally located food items and the remaining 11 (55%) chose the stimulus with food items located on the perimeter. A two-tailed binominal test revealed that the dogs’ choices were not different from chance level (*p* = 0.82).

The two observers were in 100% agreement, about both the dogs’ choices in the test trials and the number of training trials required by each dog.

### 4.3. Discussion

As in the previous experiment, dogs as a group chose the illusory arrays at chance level, indicating a lack of susceptibility to the Solitaire illusion. This finding indicates that the lack of susceptibility cannot be attributed to an insufficient knowledge or differences in salience between the stimuli. 

It is interesting to note that, even after the pre-trial, most dogs still needed several trials to learn to choose the edible item over the inedible item. Moreover, 15 dogs out of 35 were unable to learn the discrimination over 15 training trials. This indicates that dogs need training to discriminate visually similar elements from food items, at least with stimuli that are relatively unnatural, such as presentations of a single circular shaped item on a panel.

## 5. General Discussion

The current study provides evidence that dogs might not be susceptible to the Solitaire illusion. One possible reason for dogs’ lack of susceptibility is that the level of the estimation error generated by the illusion falls slightly above dogs’ quantity discrimination ratio threshold. Agrillo and colleagues [15] showed that, in humans, the illusion creates a perceived quantitative difference close to a ratio of 0.76. Data from the first experiment of the current study seem to indicate that dogs’ discriminative ratio threshold falls somewhere between 0.75—which dogs cannot discriminate—and 0.67; it is, therefore, possible that dogs’ quantity discrimination ability is not sufficiently sensitive to detect the difference generated by the spatial arrangement. On the other hand, this is not the only possible explanation, as different non-human primates show weak or no susceptibility to the illusion, regardless of their remarkably higher abilities to discriminate quantities compared with dogs [13,17].

Another possibility is that dogs’ elaboration of visual stimuli does not generate the same misrepresentations that it does in humans. The basis of humans’ susceptibility to the Solitaire illusion is believed to lie in a perceptual effect whereby identical objects located in the vicinity are perceived as a group, rather than as individual items, and are typically overestimated. In humans, perceptual grouping effects are closely linked with the global advantage of processing hierarchical stimuli [43]. Although there are limited accounts of dogs’ tendency towards perceptual grouping, one study did find a similar tendency towards global perception like humans [44], which may support the existence of perceptual grouping effects in dogs. At the same time, however, dogs’ global advantage is weaker than that of humans, and not present in all dogs [45]. Therefore, the effect may not be sufficiently strong to generate a discriminable quantity misperception. Such large individual differences in global-to-local processing have also been proposed to be the reason for dogs’ lack of susceptibility in other visual illusions based on the spatial arrangement or geometry of shapes [18,19,21,22]. Even assuming that global-to-local precedence is similar between humans and dogs, grouping mechanisms might be slightly different in the two species. In humans, the perception of a single configuration in the middle of the Solitaire array is supposed to be due to the Gestalt principles of ‘proximity’ (items that are close tend to be grouped as part of the same object) and ‘good continuation’ (items that are arranged in a straight line tend to be grouped) [12]. As dogs do not show any evidence of being susceptible to this illusion, the possibility exists that other perceptual mechanisms, e.g., similarity, closure, or common fate [12], have a larger impact on dogs’ grouping mechanisms than the ones involved in the Solitaire illusion.

The differences in quantity perception in humans and dogs are likely to reflect different underlying neurophysiological substrates. Processes of quantity assessment are generally thought to occur in the parietal cortex [46,47]. Dogs’ parietal cortex has a much simpler organization than that of primates, including only two areas, corresponding to primates’ areas 5 and 7 [46], of which the former is primarily responsible for quantitative assessments in this species [48]. Notably, area 5 is also activated during numerical tasks in primates [49], but it is not the only structure involved in quantitative assessments. In fact, the key brain area for quantification in primates is the intraparietal sulcus [46,47], which is present only in primate species. Of relevance for the present studies, recent evidence in humans showed that the degree of cortical activation in the intraparietal sulcus depends on the spatial arrangement of a set of items being viewed [47]. It is possible that the absence of a corresponding structure in dogs’ parietal cortex accounts for the species’ lack of susceptibility to the Solitaire illusion, or other arrangement-based quantitative assessment illusions.

## 6. Conclusions

We provide no evidence at group level in favour of dogs’ susceptibility to the Solitaire illusion. On the one hand, the finding may result from insufficient quantity discrimination abilities, rather than an actual lack of susceptibility to the illusion. In fact, the present study provides new evidence that dogs’ thresholds for spontaneous quantity discrimination are higher than those reported to date; nonetheless, the latter may not fall within the range of misperception created by the illusion. Considering that dogs’ quantity discrimination abilities can be improved through training [32], it would be interesting to explore their susceptibility to the illusion when trained to discriminate between quantities of higher ratios. On the other hand, combined evidence from studies on other illusions and the present findings suggest that, overall, dogs may be scarcely susceptible to quantity misperceptions linked to geometrical or spatial arrangement features of the stimuli. Owing to methodological constrains, we assessed dogs’ abilities only at the group level with no individual analyses. However, previous research with capuchin monkeys [14,17] and guppies [50] has found a large inter-individual variability, with some animals perceiving the illusion in a human-like manner and some in the opposite direction. Whether there is also an inter-individual variability with some dogs perceiving the illusion in either direction remains unexplored.
